# Biologically Active *Ajuga* Species Extracts Modulate Supportive Processes for Cancer Cell Development

**DOI:** 10.3389/fphar.2019.00334

**Published:** 2019-04-05

**Authors:** Valentin-Florian Rauca, Laurian Vlase, Tibor Casian, Alina Sesarman, Ana-Maria Gheldiu, Andrei Mocan, Manuela Banciu, Anca Toiu

**Affiliations:** ^1^Department of Molecular Biology and Biotechnology, Faculty of Biology and Geology, Babes-Bolyai University, Cluj-Napoca, Romania; ^2^Molecular Biology Centre, Institute for Interdisciplinary Research in Bio-Nano-Sciences, Babes-Bolyai University, Cluj-Napoca, Romania; ^3^Department of Pharmaceutical Technology and Biopharmacy, “Iuliu Haţieganu” University of Medicine and Pharmacy, Cluj-Napoca, Romania; ^4^Department of Pharmaceutical Botany, “Iuliu Haţieganu” University of Medicine and Pharmacy, Cluj-Napoca, Romania; ^5^Department of Pharmacognosy, “Iuliu Haţieganu” University of Medicine and Pharmacy, Cluj-Napoca, Romania

**Keywords:** *Ajuga* species, polyphenols, iridoids, antiproliferative activity, nuclear factor – kappa B, oxidative stress

## Abstract

**Backround:** *Ajuga* species have been used in traditional medicine for their diuretic, anti-inflammatory, wound-healing, and hepatoprotective properties.

**Purpose:** The phytochemical profile and anticancer potential of three *Ajuga* sp. (*A. genevensis, A. chamaepitys*, and *A. laxmannii*) from Romania was investigated.

**Materials and Methods:** The phytochemicals were extracted from the aerial parts of *Ajuga* sp. by using different solvents and methods. The hydroalcoholic extracts were examined for total phenolic, flavonoid and iridoid contents, and HPLC/MS was used to analyze the polyphenolic compounds and iridoids. The phytochemical profile was also evaluated by principal component analysis in connection with antitumor efficacy of extracts. The antiproliferative potential was evaluated using the ELISA BrdU-colorimetric immunoassay. Western Blot with regard to inflammatory protein NF-κB (nuclear factor kappa-light-chain-enhancer of activated B cells) p65 subunit expression in cell lysates was performed. Quantification of oxidative stress marker malondialdehyde (MDA) was determined by high-performance liquid chromatography (HPLC). Enzymatic and non-enzymatic antioxidant capability was assessed by measuring catalase activity and by evaluating the total antioxidant capacity (TAC) of treated cells.

**Results:** *Ajuga laxmannii* ethanol extract showed the highest total phenolic and flavonoid content, while *A. genevensis* ethanol extract was more abundant in iridoids. The overall cytostatic effect of the investigated plant extracts was exerted through strong inhibitory actions on NF-κB, the key molecule involved in the inflammatory response and via oxidative stress modulatory effects in both murine colon carcinoma and melanoma cell lines.

**Conclusion:** *Ajuga laxmannii* showed the most significant antitumor activity and represents an important source of bioactive compounds, possibly an additional form of treatment alongside conventional anticancer drugs.

## Introduction

Medicinal plants have always been an important source for various pharmaceuticals since ancient times. Nowadays the scientific interest for new drugs production from bioactive compounds isolated from natural products is still growing. Herbal medicines were often used only based on empirical observations since antiquity, without knowing the phytochemicals from the extracts or details of their pharmacological effects (Atanasov et al., 2015). Although many herbal remedies have a well-known composition and certain biological effects, some of them are still used only based on traditional medicine, and lacking the validation of their safety and efficacy. The research on unexplored medicinal plants traditionally used in folk medicine could determine the development of novel herbal formulations with significant biological activities. Due to their important pharmacological effects, the natural compounds are effectively used to obtain new phytomedicines. *Ajuga* species (Lamiaceae), which are widely distributed in many parts of the world (Atay et al., 2016) present significant medicinal importance, confirmed by the large number of constitutive compounds with anti-inflammatory, antioxidant, cytotoxic, analgesic, or antibacterial activity (Israili and Lyoussi, 2009; Toiu et al., 2018). Six *Ajuga* species are mentioned in the Romanian spontaneous flora, with *Ajuga genevensis* L. and *Ajuga reptans* L. widely distributed and used in traditional medicine, although there are few data on phytochemistry and bioactivities of Romanian species (Toiu et al., 2016, 2017). *A. laxmannii* (Murray) Benth is used in folk medicine as a galactagogue and anti-inflammatory agent. Our previous research showed the antioxidant, antimicrobial, and anti-inflammatory effects of aerial parts extracts (Toiu et al., 2018). *A. chamaepitys* (L.) Schreb. is a typical herb from Mediterranean area, which can also be found in other parts of Europe, the Near and Middle East. The monoterpene glycosides content and the essential oil composition have been recently studied on species from Italy, together with the evaluation of antioxidant activity and cytotoxicity by MTT assay (Venditti et al., 2016).

The anticancer activity of natural compounds is attributed to their synergistically acting complex mixture of phytochemicals with chemopreventive and chemotherapeutic potential, which can prove to be far more effective than isolated bioactive molecules (de Kok et al., 2008). Accordingly, the unexplored plants used in folk medicine require extensive studies for reliable evidence-based phytotherapy.

Although complementary and alternative ethnopharmacological approaches are mainly focused on counteracting the side effects and collateral symptoms of conventional cancer therapies, in this paper we investigated a potential disjunction (change in traditional plant use) (Leonti and Casu, 2013), by assessing the anticancer activity of these indigenous herbs. Therefore, this study was aimed to perform a comparative phytochemical analysis of *A. genevensis, A. chamaepitys*, and *A. laxmannii* aerial parts extracts, mainly polyphenolic compounds and iridoids, and to assess the anticancer potential against B16.F10 murine melanoma and C26 colon carcinoma cells. Both cell lines are characterized by increased metastatic potential and are prone to therapeutic alterations of their redox status (Rauca et al., 2018; Sesarman et al., 2018). In addition, melanoma and colon carcinoma are two of the deadliest cancers in modern society, possibly interlinked by epigenetic mechanisms, as recent reports concluded that colorectal cancer is one of the most common discordant cancers post-melanoma (Frank et al., 2017). As previously reported, the highly metastatic B16.F10 murine melanoma cells present a multitude of genetic alterations leading to an abnormal constitutive activation of the major signaling pathway Ras/Raf/MEK/ERK (MAPK), in contrast to normal melanocytes, in which the activation of this path is weak (Alupei et al., 2014). In colon cancer cells, as opposed to normal colon epithelial cells, the PI3K/PTEN/AKT signaling pathway is altered via frequent mutations of PTEN (Zhang et al., 2015). Moreover, one of the most important downstream targets of both Raf and AKT is NF-κB (Yajima et al., 2012), which is considered a potential key player in the treatment of melanoma (Madonna et al., 2012) and a major orchestrator in the initiation and propagation of colorectal cancer (Vaiopoulos et al., 2013). The potential synergistic interaction between bioactive constituents suggests that the whole plant extract may contribute to better therapeutic outcomes compared to the administration of single isolated compounds at an equivalent dose (Rasoanaivo et al., 2011).

## Materials and Methods

### Chemicals and Reagents

High purity chemicals: sodium carbonate, sodium acetate trihydrate, and anhydrous aluminum chloride were acquired from Sigma-Aldrich (Germany). Folin-Ciocâlteu reagent was purchased from Merck (Germany). The standard chemicals; chlorogenic acid, *p*-coumaric acid, caffeic acid, rutin, apigenin, quercetin, isoquercitrin, hyperoside, kaempferol, quercetol, myricetol, fisetin, gallic acid, aucubin, catalpol, and harpagoside were sourced from Sigma-Aldrich (Germany), ferulic acid, sinapic acid, gentisic acid, patuletin, luteolin from Roth (Germany), caftaric acid from Dalton (United States), harpagide, and 8-*O*-acetyl-harpagide from PhytoLab GmbH & Co. (Germany). HPLC grade solvents (methanol, acetonitrile, ammonium acetate, and silver nitrate) were purchased from Sigma-Aldrich. Distilled, deionised water was produced by a Direct Q-5 Millipore (Millipore SA, Molsheim, France) water system.

### Preparation of Standard Solutions

Standard stock solutions of the flavonoids and iridoids were prepared by dissolving 1 mg of each compound in 1 mL methanol and stored at 4°C, protected from daylight. They were appropriately diluted with double distilled water before being used as working solutions.

### Plant Samples and Extraction Procedures

The aerial parts of *Ajuga* species collected in flowering stage in June 2016 were obtained and authenticated by one of us (A.M.) from Cluj County, Romania and deposited in the Department of Pharmacognosy, “Iuliu Haţieganu” University, Cluj-Napoca (voucher specimens AG-23, AC-2, and AL-3, respectively). The dried plant samples were ground to a fine powder before extraction. The aerial parts extracts of *A. genevensis, A. chamaepitys*, and *A. laxmannii* were prepared by reflux extraction, using 5 g herbal material and different solvents (100 mL 70% methanol and 100 mL 70% ethanol, respectively), for 30 min, at 60°C (Methanol Extract, ME and Ethanol Extract, EE, respectively) (Toiu et al., 2018). The extracts were filtered and stored in dark glass bottle at +4°C until further analysis.

### Total Phenolic, Flavonoid, and Iridoid Content of Aerial Parts Extracts

The TPC was determined by Folin-Ciocalteu method with slight modification (Toiu et al., 2018) and expressed as GAEs, meaning mg gallic acid/g dry weight herbal material (mg GAEs/g dw) (*R*^2^ = 0.999). The TFC was determined using AlCl_3_ method (Toiu et al., 2018), and expressed as REs (mg RE/g dw) (*R*^2^ = 0.999). The TIC was determined by a photometric method based on a Trim-Hill reaction and the results were expressed as AEs (mg AE/g dw) (Erdenechimeg et al., 2017).

### High Performance Liquid Chromatography (HPLC)- Mass Spectrometry (MS) Methods

According to the previously reported methods (Vlase et al., 2012; Toiu et al., 2018) chromatographic separations were carried out on an Agilent 1100 HPLC Series system (Agilent Technologies, Darmstadt, Germany), coupled to an Agilent Ion Trap SL mass spectrometer with an electrospray or APCI ion source.

The liquid chromatograph was equipped with binary gradient pump, degasser, column thermostat and autosampler. The chromatographic separation was performed on a reversed-phase Zorbax SB-C18 (100 mm × 3.0 mm i.d., 3.5 μm) analytical column. The column temperature was set at 48°C. The chromatographic data were collected and processed by ChemStation and DataAnalysis software from Agilent, United States. The MS system operated using an electrospray ion source in negative mode. The identification and quantification of polyphenols were made in UV assisted by MS. Quantitative determinations were performed using an external standard method. In order to determine the concentration of polyphenols in plant samples, the calibration curves in the range of 0.5–50 μg/mL for a five point plot, with good linearity (*R*^2^ > 0.999) were employed. The compounds were identified by comparison of their retention times and the recorded ESI-MS with those of standards in the same chromatographic conditions (Vlase et al., 2012; Andriamadio et al., 2015). LC-ESI-MS/MS analysis of iridoids was performed using an Agilent 1100 model coupled to an Agilent Ion Trap 1100 SL MS instrument. The LC was equipped with a binary pump, autosampler, thermostat and detector (all 1100 Series from Agilent Inc., United States). The separation was carried out on an Atlantis HILIC (100 mm × 3.0 mm, 3.5 μm) analytical column. The system was controlled with Data Analysis software (version B01.03, Agilent Inc., United States).

The mass spectrometer equipped with an electrospray ionization (ESI) source operated in the positive mode, with a scan range between 360 and 680 *m/z*. The LC-ESI-MS/MS method identified the targeted iridoids (aucubin, catalpol, harpagide, harpagoside, and 8-*O*-acetyl-harpagide) based on their sodium adducts (M+23 *m/z*): aucubin (369 *m/z*), catalpol (385 *m/z*), harpagide (387.2 *m/z*), harpagoside (517.4 *m/z*) and 8-*O*-acetyl-harpagide (429.3 *m/z*), and by comparison with standards in the same chromatographic conditions. The working conditions were determined as the capillary temperature to 300°C, drying gas flow (Nitrogen) 12 L/min, and a pressure of 60 psi for the nebulizer. For quantitation of the iridoids, stock solutions of the five commercially available standards were prepared in acetonitrile. All calibration curves yielded a coefficient of determination of R^2^ ≥ 0.99. The results are expressed as μg per mL of extract (μg/mL).

### Cell Types and Culture Conditions

The B16.F10 murine melanoma cell line was cultured in DMEM medium (Lonza, Basel, Switzerland). The C26 murine colon carcinoma cell line (Cell Lines Service GmbH, Eppelheim, Germany) was cultured in RPMI-1640 medium (Lonza, Basel, Switzerland). Culture media were supplemented with 10% heat-inactivated fetal bovine serum (FBS), 100 IU/mL penicillin, 100 μg/mL streptomycin and 4 mM L-glutamine. The cells were incubated as monolayers at 37°C in a 5% CO_2_ humidified atmosphere.

### Cell Proliferation Assay

To determine the effect of *Ajuga* sp. extracts on B16.F10 murine melanoma and C26 colon carcinoma cells proliferation, 5 × 10^3^ cancer cells/well were cultured in 96-well plates for 24 h. The range of concentrations for each extract was selected based on previous studies regarding *in vitro* cytotoxic activity of *Ajuga* sp. (Sadati et al., 2012) and the effect was measured in triplicate samples for the controls (cells incubated in medium alone) and for each concentration of the vegetal extracts. To screen for ethanol toxicity, cells were incubated with the same concentrations of the solvent as those used for the preparation of the ethanolic extracts. The proliferative activity of the cancer cells after different treatments was tested using ELISA BrdU-colorimetric immunoassay (Roche Applied Science, Penzberg, Germany) as previously described (Licarete et al., 2017). Cell proliferation was calculated as percentage of untreated cells (control value). To measure the effectiveness of the treatments, the IC_50_ was calculated by GraphPad Prism version 6 for Windows software.

### Preparation of Cell Lysates

To assess the biological activity of the selected plant species, the extract concentrations that exerted strong (IC_80_) and medium (IC_50_) inhibition of proliferation in both cell lines (IC_40_ and IC_20_ in the case of EAAG on B16.F10 cells) were used for total cell lysates preparation as described previously (Licarete et al., 2017). The adherent co-cultured cells after different treatments were detached and lysed with lysis buffer containing 10 mM HEPES (pH 7), 200 mM NaCl, 1%Triton X, 10 mM MgCl_2_, 1 mM dithiothreitol (DTT), and protease inhibitor cocktail tablets (Complete, Roche Diagnostics GmbH, Germany). The homogenates were incubated for 30 min on ice and then centrifuged for 10 min at 15 000 × g, at 4°C. The supernatants were collected and stored at -80°C for molecular investigations (Rauca et al., 2018). To determine the protein concentration of each sample, the Bradford assay was used (BioRad, Hercules, CA, United States) (Alupei et al., 2014).

### Western Blot Analysis of the Expression Levels of NF-κB-p65 Subunit

To assess the effect of the selected ethanolic extracts on the expression of key inflammatory transcription factor NF-κB-p65 subunit in the cell lysates obtained from standard C26 and B16.F10 cell culture, western blot analysis was performed, as previously described (Patras et al., 2016). A total of 40 μg of total protein was loaded per lane onto a 10% polyacrylamide gel. Electrophoresis was performed at 50 mV, and the electro-transfer of proteins onto a nitrocellulose membrane was conducted at 100 mV for 50 min. The membranes were blocked with 5% skimmed milk powder (Bio-Rad Laboratories, Inc., Hercules, CA, United States) in TBS containing 0.1% Tween-20 (TBS-T) for 3 h at room temperature. Subsequently, the membranes were incubated at 4°C overnight with mouse monoclonal anti-NF-κB-p65 antibodies (sc56735; Santa Cruz Biotechnology, Inc., Dallas, TX, United States), diluted 500-fold in 5% skimmed milk powder in TBS-T. A primary rabbit polyclonal antibody against mouse β-actin (A2103; Sigma-Aldrich; Merck KGaA), diluted 1,000-fold in TBS-T, was used for the loading control. To detect the bound antibodies, the membranes were washed with TBS-T and incubated with a goat anti-rabbit (sc-2004; Santa Cruz Biotechnology, Inc.) or a goat anti-mouse horseradish peroxidase (HRP)-conjugated IgG (secondary antibody, sc-2005; Santa Cruz Biotechnology, Inc.), diluted 4,000-fold in 5% skimmed milk powder in TBS-T at room temperature for 1 h. The detection was performed by using Clarity^TM^ Western ECL kit (Bio-Rad Laboratories, Hercules, CA, United States) and the membranes were exposed to an X-ray film (Kodak, Knoxville, TN, United States) for 3 min max. The analysis of the films was performed using Image J freeware for Windows 7 64 bit (Licarete et al., 2017). The final results were presented as mean ± standard deviation (SD) of two independent experiments.

### Measurement of Oxidative Stress Parameters

Malondialdehyde levels in cell lysates were determined by high-performance liquid chromatography (HPLC) as previously described (Licarete et al., 2017). After cell lysates deproteinization, quantification of MDA was performed using HPLC column type RP18 (5 μm) (Supelco, Bellefonte, PA, United States) and a mobile phase consisting of 30 mM KH_2_PO_4_/methanol in a volume ratio of 65:35. Flow rate was set at 1 mL/min and MDA absorbance was measured at 254 nm. The retention time of MDA was about 2.8 min. Data were normalized to the protein concentration from the cell lysates and expressed as μg MDA/mg protein. Each sample was determined in duplicate. The measurement of catalase activity was performed according to the method of Aebi and was expressed as units of catalytic activity/mg of protein (Aebi, 1984). The assessment of TAC was based on the method described by Erel and the results expressed as mmoles of non-enzymatic antioxidants/mg of protein (Erel, 2004). The samples were measured in duplicate.

### Multivariate Data Analysis

The interspecies induced variability in the phytochemical composition of extracts was evaluated using OPLS-DA (Orthogonal PLS – Discriminant Analysis). Prior to model development the X dataset, represented by the phytochemical composition of each extract, and Y dataset, represented by a binary variable matrix encoding class membership were scaled to unit variance. Class membership of observations was assigned in function of plant species. Model performance was evaluated through the fraction of explained variability by each component (R2X), the total variation of Y explained by the model (R2Y), and predictive capacity (Q2) calculated using full cross-validation. Interpretations were done by generating the corresponding score and loading plots (SIMCA 15, Sartorius Stedim, Sweden).

Correlations between biological activity and extract type were evaluated using PLS method, through an experimental design approach (Modde 11 Pro, Sartorius Stedim, Sweden). The full factorial experimental design was built on three factors, namely extract type (EEAG, EEAC, and EEAL), cell type (B16F10, C26), and extract concentration. The response was represented by the percentage of proliferation inhibition against the control group (untreated cells). Model performance was evaluated using R2, Q2, Validity and Reproducibility parameters, while model significance and lack of fit were assessed using F-testing. Interpretations were done by generating coefficient plots, and the significance of each coefficient (factor) was tested using ANOVA.

### Statistical Analysis

All phytochemical assays were performed in triplicate, and the results were expressed as the mean ± S.D. Statistical comparisons between two independent groups were performed using the Student’s *t*-test (with equal and unequal variances, depending upon to the results of the F-test) for normally distributed data. Pearson and Spearman’s correlation analyses were used to calculate statistical relationships between parameters. Analyses were performed using SPSS 16.0 for Windows (SPSS Inc., United States). Data from different experiments were indicated as mean ± SD. The IC_20_, IC_40_, IC_50_, and IC_80_ values of different treatments were calculated by using non-linear regression of sigmoidal dose response curves and interpolation of fit offered by the GraphPad Prism version 6 for Windows, GraphPad Software (San Diego, CA, United States). Phytochemical composition of extracts originating from different species was evaluated using OPLS-DA (Orthogonal PLS – Discriminant Analysis). The antioxidant and anti-inflammatory effects of the selected *Ajuga* sp. extracts on C26 and B16.F10 cell lines were evaluated using one-way ANOVA with Bonferroni correction for multiple comparisons. A value of *P* < 0.05 was considered statistically significant.

## Results

### Total Bioactive Compounds and HPLC-MS Analysis

Results for TPC, TFC, and TIC for the *Ajuga* sp. extracts are presented in Table 1. In all cases the ethanol extracts contained higher amounts of each class of bioactive compounds compared to methanol extracts. The values for TPC and TFC were higher in EEAL extracts, followed by EEAC, whereas EEAG extract was more abundant in iridoids. The content in total iridoids was similar in *A. genevensis* and *A. laxmannii* extracts. The results were in good agreement with our previous studies on EEAL extracts (Toiu et al., 2018).

**Table 1:** Total phenolic, flavonoid, and iridoid content in *A. genevensis* (AG), *A. chamaepitys* (AC), and *A. laxmannii* (AL) extracts.

Extract	Total phenolic content (mg GAEs/g dw)		Total flavonoid content (mg REs/g dw)		Total iridoid content (mg AEs/g dw)	
	ME	EE	ME	EE	ME	EE
AG	20.63 ± 0.78	24.39 ± 0.73	10.96 ± 0.83	12.71 ± 0.69	17.39 ± 0.65	18.22 ± 0.86
AC	42.59 ± 0.86	46.61 ± 0.9	18.49 ± 0.58	20.05 ± 0.63	10.61 ± 0.48	11.5 ± 0.51
AL	61.35 ± 0.97	68.77 ± 1.48	32.19 ± 0.41	37.23 ± 0.68	15.09 ± 0.72	16.11 ± 0.62

*Data are shown as mean ± SD of triplicate measurements*.

**Figure 1:**
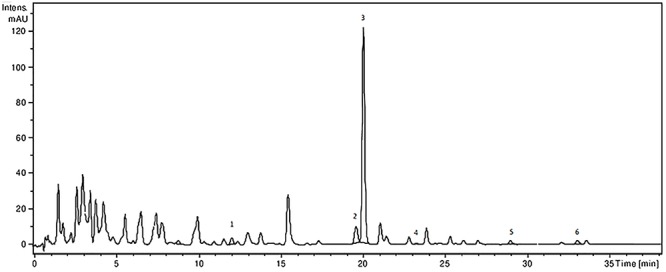
HPLC-UV-MS chromatogram of polyphenolic compounds from *A. laxmannii* ethanol extract. The identified compounds: ferulic acid (1), isoquercitrin (2), rutin (3), quercitrin (4), luteolin (5), and apigenin (6).

**Figure 2:**
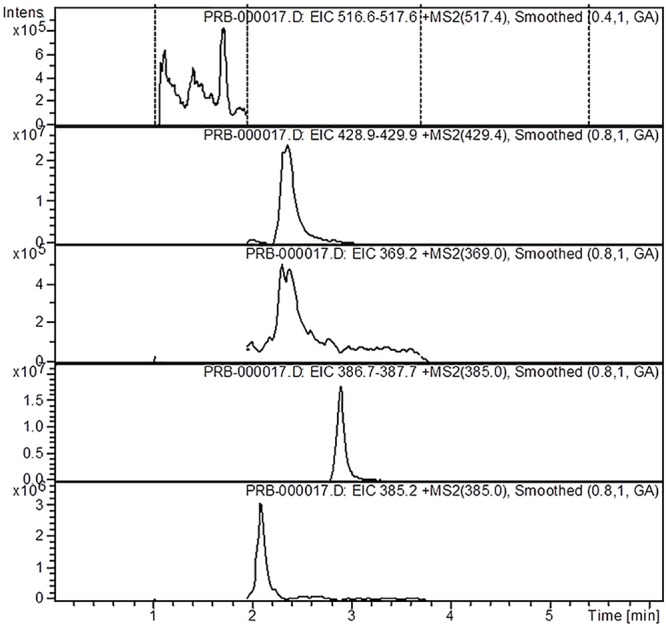
HPLC-MS-MS chromatogram of iridoids from *A.genevensis* ethanolic extract.

**Figure 3:**
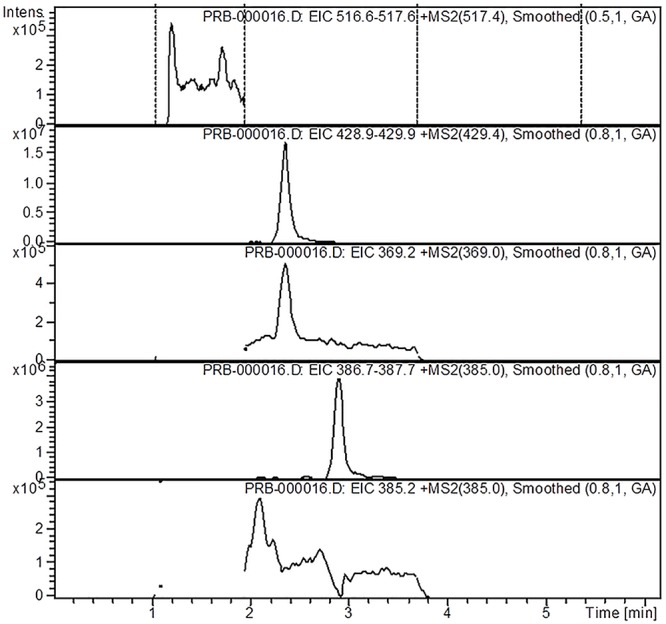
HPLC-MS-MS chromatogram of iridoids from *A. chamaepitys* ethanol extract.

The analysis of the *Ajuga* sp. extracts using an optimized HPLC/MS method showed the presence of 9 polyphenols (three phenolcarboxylic acids, four flavonoid glycosides, and two free aglycones) (Figure 1 and Table 2) The HPLC-MS analysis of iridoids indicated that the major compound identified in all extracts was 8-*O*-acetyl-harpagide, followed by harpagide, while the aucubin and catalpol were found in lower amount (Figure 2, 3 and Table 3). The spectrophotometric determinations revealed that EEAG contains the highest amount of iridoids, and the HPLC analysis showed the same tendency. The obtained results allow the characterization of *Ajuga* sp. extracts in main biologically active compounds, therefore the possibility of correlation between the therapeutic effect and the dose.

**Table 2:** Polyphenolic profile of *A. genevensis* (AG), *A. chamaepitys* (AC), and *A. laxmannii* (AL) extracts.

	Compound content (μg/g dw)					
	EEAG	MEAG	EEAC	MEAC	EEAL	MEAL
Caffeic acid	25.37 ± 1.01	21.26 ± 1.05	ND^∗^	ND	ND	ND
*p*-Coumaric acid	24.31 ± 1.15	14.83 ± 1.09	ND	ND	ND	ND
Ferulic acid	18.49 ± 0.27	18.13 ± 0.31	53.65 ± 2.18	42.81 ± 2.09	25.32 ± 1.55	21.39 ± 1.43
Hyperoside	7.23 ± 0.09	6.57 ± 0.16	ND	ND	ND	ND
Isoquercitrin	ND	ND	180.77 ± 2.84	151.1 ± 2.77	689.22 ± 4.95	638.5 ± 6.01
Rutin	ND	ND	9.02 ± 0.44	7.11 ± 0.31	6879.23 ± 8.82	6718.39 ± 8.75
Quercitrin	16.37 ± 0.19	12.25 ± 0.16	36.89 ± 1.55	34.54 ± 1.31	43.66 ± 1.73	38.81 ± 1.59
Luteolin	45.24 ± 1.86	41.83 ± 1.75	228.41 ± 3.76	215.58 ± 3.67	120.36 ± 1.1	86.29 ± 1.03
Apigenin	27.61 ± 1.52	24.42 ± 1.31	ND	ND	128.59 ± 2.42	125.45 ± 2.29

^∗^*ND-not determined. Data are shown as mean ± SD of triplicate measurements*.

**Table 3:** Iridoid profile of *A. genevensis* (AG), *A. chamaepitys* (AC), and *A. laxmannii* (AL) extracts.

	Compound content (μg/mL)					
	EEAG	MEAG	EEAC	MEAC	EEAL	MEAL
Harpagide	195.5 ± 3.44	190.6 ± 4.8	160.2 ± 3.28	156.8 ± 3.12	114.5 ± 2.87	110.3 ± 2.54
Aucubin	7.4 ± 0.67	6.9 ± 0.68	6.2 ± 0.59	5.7 ± 0.52	4.3 ± 0.4	4.03 ± 0.25
Catalpol	10.3 ± 1.41	9.7 ± 1.47	8.5 ± 1.24	7.9 ± 1.11	6.2 ± 0.32	5.3 ± 0.28
Harpagoside	1.1 ± 0.11	0.9 ± 0.06	0.9 ± 0.07	0.7 ± 0.05	35.1 ± 2.08	28.4 ± 1.72
8-*O*-acetyl-harpagide	477.3 ± 5.16	471.5 ± 5.02	393.7 ± 4.15	387.3 ± 4.09	280.5 ± 3.31	277.4 ± 3.08

*Data are shown as mean ± SD of triplicate measurements*.

### Antiproliferative Activity of *Ajuga* sp. Extracts on C26 and B16.F10 Cancer Cell Lines

**Figure 4:**
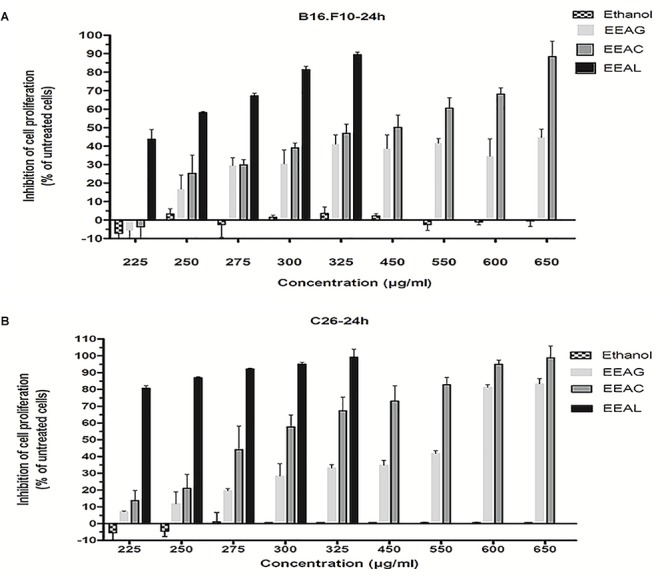
Effects of *Ajuga* sp. extracts on cell proliferation. **(A)** 24 h after incubation of B16.F10 cells with different concentrations of EEAG, EEAC, and EEAL extracts. **(B)** 24h after incubation of C26 cells with different concentrations of EEAG, EEAC, and EEAL extracts. Data are shown as mean ± SD of triplicate measurements. EEAG, *A. genevensis* ethanolic extract; EEAC, *A. chamaepitys* ethanolic extract; EEAL, *A. laxmannii* ethanolic extract. Ethanol-treated cells were used as toxicity controls.

**Figure 5:**
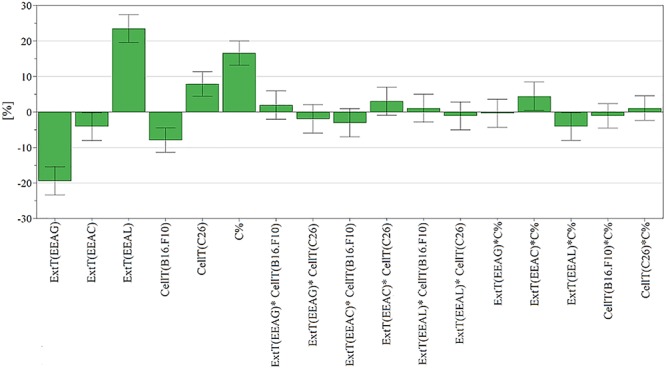
Scaled and centered coefficient bar plot for cell proliferation inhibition. To analyze the effect of factors on cell proliferation inhibition, the coefficients of the polynomial equation were plotted. The coefficient of a factor represents the change in response induced by increasing the factor’s level from low to high while all other factors are at average level. Each coefficient has a 95% confidence interval, represented through error bars, and reflecting its uncertainty. EEAG, *A. genevensis* ethanolic extract; EEAC, *A. chamaepitys* ethanolic extract; EEAL, *A. laxmannii* ethanolic extract.

The effects of different treatments at various concentrations (50–650 μg/mL) on the proliferation of C26 and B16.F10 cells were expressed as percentage of inhibition compared to the proliferation of the untreated control cells (Figure 4A,B) and as IC_50_ values for each extract tested (Table 4). Our data showed that EEAL exerted strong inhibitory effects at much lower concentrations than EEAC and EEAG on B16.F10 melanoma (Figure 4A and Table 4) as well as on C26 Colon Carcinoma Cells (Figure 4B and Table 4). The relationship between input variables (plant species, extract concentration, cell type – X dataset) and cell proliferation inhibition rate (Y dataset) was assessed by fitting a polynomial equation through PLS method (Figure 5). The specific types of polyphenols (isoquercitrin, rutin and apigenin) (Table 2) and iridoids (harpagoside and 8-*O*-acetyl-harpagide) (Table 3) might be involved in strong antitumor activity of the vegetal extracts tested.

**Table 4:** Cytotoxicity of *Ajuga* sp. ethanolic extracts against C26 and B16.F10 murine cancer cell lines by ELISA BrdU-colorimetric immunoassay (IC_50_ value, μg/mL).

Cell line	C26		B16.F10	
Treatment	IC_50_	Confidence interval 95%	IC_50_	Confidence interval 95%
EEAG (*A. genevensis*)	457.5	374.0–559.7	741.4	388.5–1415
EEAC (*A. chamaepitys*)	303.0	274.8–334.1	406.7	341.7–484.1
EEAL (*A. laxmannii)*	176.3	154.5–201.1	236.8	227.1–246.8

*IC_*50*_ represents the half maximal inhibitory concentration for the tested drugs*.

### Strong Inhibitory Actions of the Vegetal Extracts on NF-κB-p65 Expression in C26 and B16.F10 Total Cell Lysates

**Figure 6:**
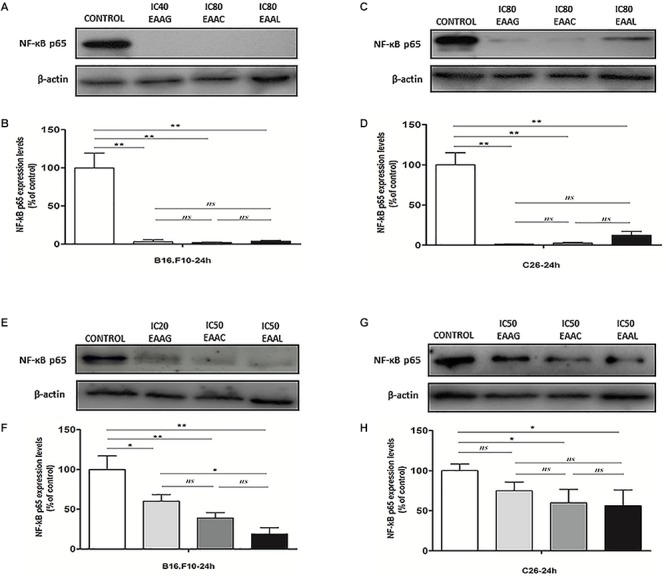
The expression of NF-κB-p65 in cell lysates after different treatments. Western blot analysis of total NF-κB-p65 expression in cell lysates from **(A,E)** B16.F10 cells and **(C,G)** C26 cells after different treatments; β-actin was used as loading control. **(B)** % of NF-κB-p65 expression relative to control in B16.F10 melanoma cells after IC_40_ EEAG, IC_80_ EEAC, and IC_80_ EEAL treatments. **(F)** % of NF-κB-p65 expression relative to control in B16.F10 melanoma cells after IC_20_ EEAG, IC_50_ EEAC, and IC_50_ EEAL treatments. **(D)** % of NF-κB expression relative to control in C26 colon carcinoma cells after IC_80_ EEAG, IC_80_ EEAC, and IC_80_ EEAL treatments. **(H)** % of NF-κB expression relative to control in C26 colon carcinoma cells after IC_50_ EEAG, IC_50_ EEAC, and IC_50_ EEAL treatments. On B16.F10 cells: Control, untreated cells; IC_40_ or IC_20_ EEAG, cells incubated with 650 μg/mL or 260 μg/mL *A. genevensis* ethanolic extract; IC_80_ or IC_50_ EEAC, cells incubated with 650 μg/mL or 406.7 μg/mL *A. chamaepitys* ethanolic extract; IC_80_ or IC_50_ EEAL, cells incubated with 325 μg/mL or 236.8 μg/mL *A. laxmannii* ethanolic extract. On C26 cells: Control, untreated cells; IC_80_ or IC_50_ EEAG, cells incubated with 650 μg/mL or 457.5 μg/mL *A. genevensis* ethanolic extract; IC_80_ or IC_50_ EEAC, cells incubated with 650 μg/mL or 303 μg/mL *A. chamaepitys* ethanolic extract; IC_80_ or IC_50_ EEAL, cells incubated with 325 μg/mL or 176.3 μg/mL *A. laxmannii* ethanolic extract. Results represent the mean ± SD of two independent measurements. One way ANOVA test with Bonferroni correction for multiple comparisons was used to analyze the effects of different treatments on the levels of NF-κB-p65 in comparison with the pro-inflammatory transcription factor production in control (ns, *P* > 0.05; ^∗^*P* < 0.05;^∗∗^*P* < 0.01).

Our results indicated that IC_80_ concentrations of all *Ajuga* sp. extracts tested in this study (IC_40_ in the case of EEAG tested on B16.F10) elicited a very strong inhibition (≥80% compared with control) of the key inflammatory transcription factor NF-κB-p65 expression (Figure 6A–D). In addition, the IC_50_ concentrations (Table 4) tested on both cell lines (IC_20_ in the case of EEAG tested on B16.F10) determined various levels of inhibition (30–70% compared with control) (Figure 6E–H).

### Modulatory Effects of *Ajuga* sp. Extracts on “Physiological” Oxidative Stress of Cancer Cells

**Figure 7:**
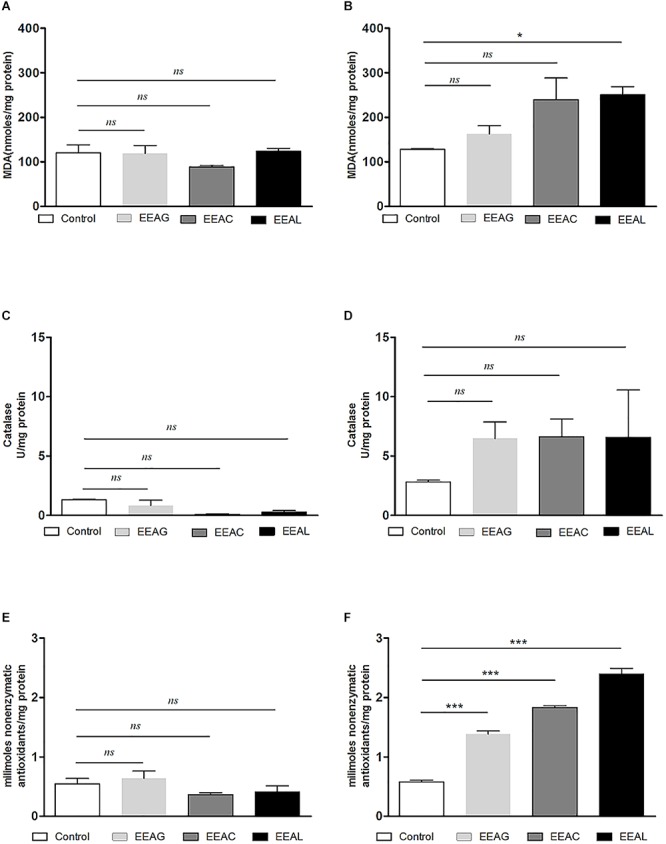
Effects of *Ajuga* sp. extracts on the oxidative stress generated by C26 colon carcinoma cells. **(A,B)** Malondialdehyde (MDA) concentration after **(A)**: IC_50_ EEAG, IC_50_ EEAC, and IC_50_ EEAL treatment and **(B)**: IC_80_ EEAG, IC_80_ EEAC, and IC_80_ EEAL treatment. **(C,D)** Catalytic activity of catalase after **(C)**: IC_50_ EEAG, IC_50_ EEAC, and IC_50_ EEAL treatment, and **(D)**: IC_80_ EEAG, IC_80_ EEAC, and IC_80_ EEAL treatment. **(E,F)** Total non-enzymatic antioxidant system levels in the cell lysates obtained from standard culture of C26 colon carcinoma cells after 24 h of incubation with **(E)**: IC_50_ EEAG, IC_50_ EEAC, and IC_50_ EEAL treatment, and **(F)**: IC_80_ EEAG, IC_80_ EEAC, and IC_80_ EEAL treatment. One way ANOVA test with Bonferroni correction for multiple comparisons was performed to analyze the differences between the effects of the treatments applied on MDA and non-enzymatic antioxidant defense systems levels and on catalase activity. Control, untreated C26 cells; IC_80_ or IC_50_ EEAG, cells incubated with 650 μg/mL or 457.5 μg/mL *A. genevensis* ethanolic extract; IC_80_ or IC_50_ EEAC, cells incubated with 650 μg/mL or 303 μg/mL *A. chamaepitys* ethanolic extract; IC_80_ or IC_50_ EEAL, cells incubated with 325 μg/mL or 176.3 μg/mL *A. laxmannii* ethanolic extract. (ns, *P* > 0.05; ^∗^*P* < 0.05; ^∗∗∗^*P* < 0.001).

**Figure 8:**
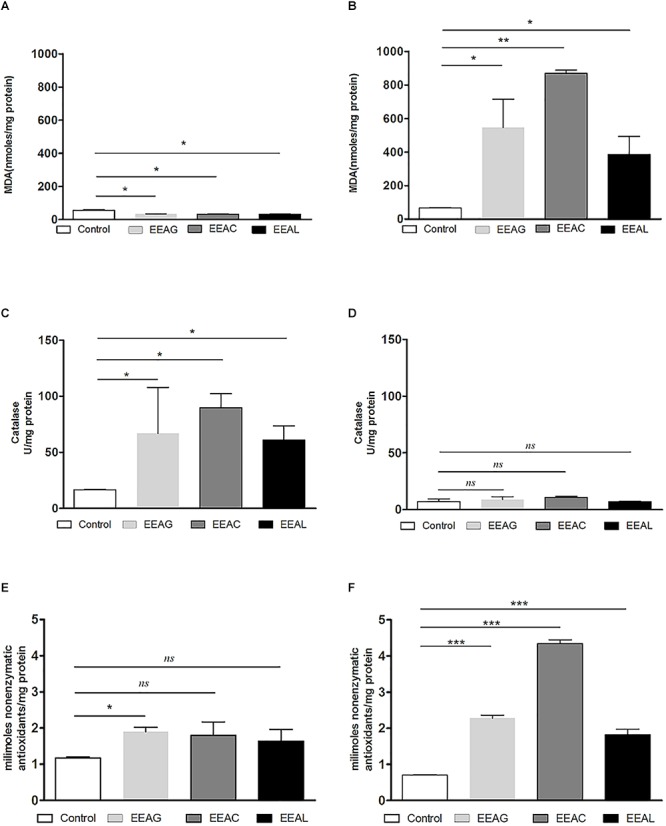
Effects of *Ajuga* sp. extracts on the oxidative stress generated by B16.F10 melanoma cells. **(A,B)** MDA concentration after **(A)**: IC_20_ EEAG, IC_50_ EEAC, and IC_50_ EEAL treatment and **(B)**: IC_40_ EEAG, IC_80_ EEAC, and IC_80_ EEAL treatment. **(C,D)** Catalytic activity of catalase after **(C)** IC_20_ EEAG, IC_50_ EEAC, and IC_50_ EEAL treatment and **(D)**: IC_40_ EEAG, IC_80_ EEAC, and IC_80_ EEAL treatment. **(E,F)** Total non-enzymatic antioxidant system levels in the cell lysates obtained from standard culture of B16.F10 murine melanoma cells after 24 h of incubation with **(E)**: IC_20_ EEAG, IC_50_ EEAC, and IC_50_ EEAL treatment and **(B)**: IC_40_ EEAG, IC_80_ EEAC, and IC_80_ EEAL treatment. One way ANOVA test with Bonferroni correction for multiple comparisons was performed to analyze the differences between the effects of the treatments applied on MDA and non-enzymatic antioxidant defense systems levels and on catalase activity. Control, untreated B16.F10 cells; IC_40_ or IC_20_ EEAG, cells incubated with 650 μg/mL or 260 μg/mL *A. genevensis* ethanolic extract; IC_80_ or IC_50_ EEAC, cells incubated with 650 μg/mL or 406.7 μg/mL *A. chamaepitys* ethanolic extract; IC_80_ or IC_50_ EEAL, cells incubated with 325 μg/mL or 236.8 μg/mL *A. laxmannii* ethanolic extract. (ns, *P* > 0.05; ^∗^*P* < 0.05; ^∗∗^*P* < 0.01; ^∗∗∗^*P* < 0.001).

As cancer cells are under persistent oxidative stress (Alupei et al., 2015) we investigated the potential relationship between the antiproliferative activity of the vegetal extracts, and oxidative stress generated in both cancer cell types. Thus, the levels of a general oxidative stress marker – MDA, as well as the catalytic activity of catalase and production of non-enzymatic antioxidant systems were assessed on both cell lines and are shown in Figure 7, 8. Our results indicated that the IC_80_ concetrations of the extracts (IC_40_ in the case of EEAG on B16.F10) increased the pro-oxidative damage (Figure 7B, 8B) in correlation with a proportional increase in the antioxidant capacity of the remaining cancer cells (Figure 7F, 8F) of both cell lines. The activity of the antioxidant enzyme catalase on both cell lines was not significantly modified by the IC_80_ extract concentrations used in this investigation (Figure 7D, 8D). However, the IC_50_ extract concentrations (IC_20_ in the case of EEAG) had a moderate antioxidant effect on B16.F10 cells by reducing the levels of MDA compared to control (Figure 8A) and slightly stimulating catalase activity (Figure 8C). On C26 cells, the IC_50_ extract concentrations did not significantly modify any of the parameters of oxidative stress tested (Figure 7A,C,E).

### Multivariate Data Analysis

**Figure 9:**
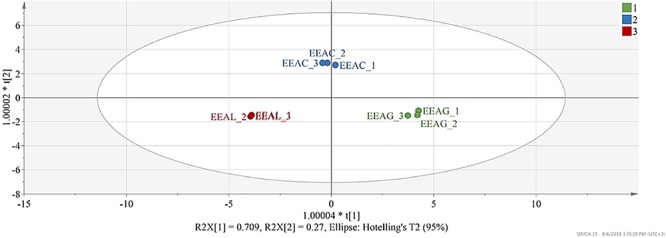
Score scatter plot of the OPLS-DA model (R2Y-99.8% and Q2-99.5%) – t(1) – first predictive component vs. t(2) – second predictive component. Each observation is represented by a point in multidimensional space, differentially colored according to plant species. EEAG, *A. genevensis* ethanolic extract; EEAC, *A. chamaepitys* ethanolic extract; EEAL, *A. laxmannii* ethanolic extract. R2X-fraction of variability in X (physicochemical descriptors) explained by each component; R2Y-total variation of Y (class membership) explained by the model; Q2-predictive capacity.

The model built to analyze the phytochemical composition of ethanolic extracts of the three different species consisted of two predictive components and one orthogonal component. 96% of X variation captured by two predictive components was found correlated with class separation, while only 2.16% variability was attributed as orthogonal. Both predictive components were found significant judging from their eigenvalues, first predictive component presented an eigenvalue of 11.8 (R2X-69.4%) and the second predictive component 4.56 (R2X-26.8%). From the score scatter plot presented under Figure 9, a good separation of observations can be seen in function of class membership, reflected also in the total sum of variation in Y explained by the model (R2Y- 99.5%) and in the goodness of prediction (Q2-99.1%).

**Figure 10:**
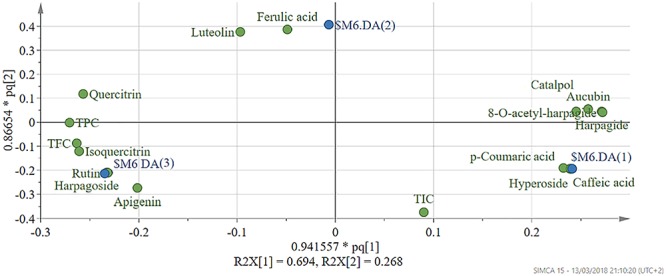
Loading scatter plot of OPLS-DA model – pq(1) vs. pq(2). Physicochemical descriptors in relation to the artificial dummy variables that reflect class membership: $M6.DA(1)-EEAG; $M6.DA(2)-EEAC; $M6.DA(3)-EEAL. p- X loading weight; q- Y loading weight. EEAG, *A. genevensis* ethanolic extract; EEAC, *A. chamaepitys* ethanolic extract; EEAL, *A. laxmannii* ethanolic extract.

To identify the phytochemical descriptors responsible for class separation the corresponding loading plot was generated (Figure 10). Graphical interpretation of the loading plot is made by considering the positioning of physicochemical descriptors in relation to the artificial dummy variables that reflect class membership [$M6.DA(1), $M6.DA(2), and $M6.DA(3)]. The right hand-group of variables represented by caffeic acid, *p*-coumaric acid, hyperoside, 8-*O*-acetyl-harpagide, harpagide, aucubine and catalpol are found in higher concentration in extract EEAG [situated close to $M6.DA(1)] and in lower concentration in extracts EEAL [situated in opposite direction from $M6.DA(3)], while EEAC has middle concentrations in these phytochemical descriptors. In a similar way, the left hand-group of variables represented by rutin, harpagoside, apigenin, isoquercitrin, quercitrin are found in higher concentration in extracts EEAL, respectively in lower concentration in extract EEAG and intermediate concentration in extract EEAC. TPC and TFC parameters are correlated with the left hand-group variables, presenting higher values for extracts EEAL. The second predictive component captures variability related to ferulic acid and luteolin content, respectively TIC. EEAG extracts presented higher content of ferulic acid, luteolin and low values of TIC, while EEAC and EEAL extracts show an opposite pattern.

The relationship between input variables (plant species, extract concentration, cell type – X dataset) and cell proliferation inhibition rate (Y dataset) was assessed by fitting a polynomial equation through PLS method. The developed model explained most of the response variation (R2-0.85) and had good predictive capacity (Q2-0.71) and high reproducibility (0.953). Comparing the modeled response variation with un-modeled part (residuals) yielded a significant model, *p* < 0.05 (5.59e–17). The residuals were further decomposed into model error and replicate error and compared. According to *p* value of 0.146 (*p* > 0.05) the replicate and model error originate from the same distribution, meaning there is no lack of fit. The model validity parameter (0.518) calculated using the lack of fit *p* value was above 0.25, also suggesting a valid model (Surowiec et al., 2015). To analyze the effect of factors on cell proliferation inhibition, the coefficients of the polynomial equation were plotted (Figure 5). The coefficient of a factor represents the change in response induced by increasing the factor’s level from low to high while all other factors are at average level. Each coefficient has a 95% confidence interval, represented through error bars, reflecting its uncertainty (Casian and Iurian, 2017).

## Discussion

In the present study, we evaluated the antiproliferative potential of three indigenous *Ajuga* sp. extracts, EEAG (*A. genevensis*), EEAC (*A. chamaepitys*), and EEAL (*A. laxmannii*) on C26 and B16.F10 murine cancer cell lines via ELISA BrdU-colorimetric immunoassay. Additionally, we investigated the potential anti-inflammatory and antioxidant/pro-oxidant activity underlying the observed cancer cell growth inhibition. As shown in Figure 4A,B, standardized ethanolic extracts of the three selected species induced different degrees of proliferation inhibition in both cell lines after 24 h. EEAL exhibited the strongest inhibitory effect with a corresponding IC_50_ of 176.3 μg/mL on C26 cells and 236.8 μg/mL on B16.F10 cells (Table 4). To analyze the effect of factors on cell proliferation inhibition, the coefficients of the polynomial equation were plotted. According to the coefficient plot (Figure 5), the most important factor affecting cell proliferation was the plant species, followed by extract concentration and cell type. EEAL exerted the best biological activity, inhibiting the proliferation of both cell lines most efficiently, while EEAG yielded the lowest inhibition levels. Independent of plant type, the exerted effects were concentration dependent, higher concentrations being more effective. Regarding the type of cell culture, higher inhibition rates were obtained for C26 compared to B16.F10 cells. The absence of significant interactions between extract type and cell type suggests that for all extracts, this cell type dependent proliferation inhibition has the same pattern. The differences in biological activity of extracts originating from the three *Ajuga* sp. were consistent with the good separation of observations in function of class membership (Figure 9) and further interpretation by OPLS-DA enabled the identification of phytochemical compounds responsible for the increased antiproliferative activity of EEAL. Variables situated in the left hand-group of Figure 10 (rutin, harpagoside, apigenin, isoquercitrin, quercitrin, TPC, and TFC) are responsible for the increased biological activity of EEAL extract. This was in concordance with previous studies revealing that isoquercitrin suppresses colon cancer cell growth *in vitro* (Amado et al., 2014) and that rutin, quercitrin and isoquercitrin tested separately present different degrees of antiproliferative effects (Orfali et al., 2016). Moreover, apigenin administered alone on cancer cells inhibited proliferation, invasion and migration (Yan et al., 2017). Synergistic antiproliferative effects demonstrated in cancer cell lines provided with flavonoid combination treatments further support our findings (Campbell Jessica et al., 2006). The same biologically active compounds were found in lower concentration in EEAG and intermediate concentration in EEAC, also reflected in the different degrees of proliferation inhibition (Figure 4A,B). EEAC extract presented higher content of ferulic acid, luteolin and low values of TIC, while EEAG and EEAL showed an opposite pattern (Figure 10). Luteolin’s antiproliferative activity resides in the potential to induce cell cycle arrest via inhibiting IGF-1-mediated PI3K/PKB activation (Tuorkey, 2016), and ferulic acid has been proven effective in inhibiting proliferation of osteosarcoma cells (Zhang et al., 2016). The right hand-group of variables represented by caffeic acid, *p*-coumaric acid, hyperoside, 8-*O*-acetyl-harpagide, harpagide, aucubine and catalpol (Figure 10) are found in higher concentration in extract EEAG and in lower concentration in extract EEAL, while EEAC has middle concentrations in these phytochemical descriptors. Among these bioactive compounds, caffeic acid, and *p*-coumaric acid can decrease cell proliferation and attenuate the viability of cancer cells *in vitro* (Anantharaju et al., 2016).

The pattern of polyphenols indicates large differences between the three *Ajuga* sp., therefore they could be used as potential taxonomic markers. Luteolin was found in highest amount in EEAC. The major compound from EEAL was rutin, in high yield, whilst in EEAC it was present in small quantities and in EEAG was not determined. Rutin had been previously reported by several *in vitro* and *in vivo* studies for the anti-inflammatory, antioxidant, neuroprotective, nephroprotective, hepatoprotective, and antihyperglycemic effects (Ghorbani, 2017). However, other studies highlighted the fact that flavonoids such as rutin and quercetin derivatives can act as pro-oxidant molecules via auto-oxidation of the hydroxyl group at the 3 position, depending on concentration and reaction conditions (Kessler et al., 2003). Accordingly, our experimental data reported what might be a dose dependent “double-edged sword” effect of *Ajuga* sp. extracts on B16.F10 melanoma cell generated oxidative stress. Lower doses (IC_20_ EEAG, IC_50_EEAC, and IC_50_ EEAL) had a moderate anti-oxidant effect by slightly reducing MDA levels and stimulating catalase activity (Figure 8A), while higher doses (IC_40_ EEAG, IC_80_ EEAC, and IC_80_ EEAL) increased MDA levels in correlation with a proportional increase in the total anti-oxidant capacity of the cancer cells. This indicates that melanoma cells might become resistant to high doses of *Ajuga* sp. extracts because of their ability to increase the levels of endogenous antioxidants and melanin production (Denat et al., 2014; Rauca et al., 2018), as an adaptive response to oxidative damage. In the case of C26 colon carcinoma cells, lower doses (IC_50_ EEAG, IC_50_ EEAC, and IC_50_ EEAL) did not significantly modulate the oxidative stress parameters (Figure 7A,C,D). The higher doses (IC_80_ EEAG, IC_80_ EEAC, and IC_80_ EEAL) revealed an overall anti-oxidant effect suggested by a strong increase in the total anti-oxidant capacity of the cells (Figure 7F) and the absence of a significant MDA level increase by EEAG and EEAC treatment, compared to a moderate MDA level increase by EEAL treatment (Figure 7B). The differences between the extract effects on the two cancer cell lines might also be explained by the higher IC_50_ values on B16.F10 cells compared to IC_50_ values on C26 cells (Table 4). Only in the case of EEAL, effective at lower concentrations, the pro-oxidant state can be correlated with anti-proliferative effects on both cell lines, as previous studies suggested that, depending on the cellular context, even though cancer cell survival can rely on antioxidant activity, high doses of an agent with antioxidant properties can effectively kill the cells by enhancing ROS production (Hawk et al., 2016). Thus, EEAG and EEAC effective at higher concentrations determined higher levels of MDA on B16.F10 cell line, close to the upper limit of physiological range (μM), which can directly kill cancer cells. This finding is consistent with previous studies, showing that cancer cells are more susceptible to ROS and phenolic compounds can act as pro-oxidants under certain conditions, such as high concentrations (Leon-Gonzalez et al., 2015). Based on the proliferation results, the extract doses that exerted strong inhibitory effects (IC_80_) and moderate inhibitory effects (IC_50_, IC_40_, and IC_20_) were used to further investigate the molecular mechanisms of cancer cell growth inhibition. Constitutive expression of NF-κB-p65, the pivotal transcription factor in inflammation and tumor cell proliferation (Xia et al., 2014) was strongly inhibited by the higher concentrations (IC_80_/IC_40_) of all tested extracts on both cell lines (Figure 6A–D) and presented a moderate to strong inhibition in the cells treated with IC_50_/IC_20_ concentrations (Figure 6E–H). The importance of NF-κB-p65 levels of expression in the outcome of different types of cancer was previously demonstrated (Weichert et al., 2007; Zhu et al., 2017), concluding that either nuclear or cytoplasmic expression of the transcription factor was correlated with unfavorable prognosis of solid tumors (Wu et al., 2015). Moreover, the high constitutive levels can be correlated with a higher constitutive activation of NF-κB in melanoma (Madonna et al., 2012), as well as in colon carcinoma, reported by our previous studies (Luput et al., 2018; Sesarman et al., 2018). The constitutive activation of NF-κB in several types of cancer regulates cell proliferation, apoptosis, and cell migration (Karin et al., 2002). Thus, the reduced NF-κB-p65 expression levels (Figure 6A–H) might contribute to the cytostatic effect of the investigated plant extracts. Although *in vivo* NF-κB tumor-specific suppression on melanoma is beneficial, generalized suppression of NF-κB is harmful (Enzler et al., 2011). The almost complet reduction of NF-κB-p65 expression levels at high concentrations (Figure 6B,D) indicates potential host toxicity, therefore, only the IC_50_ values (IC_20_ in the case of EEAG on B16.F10) (Figure 6F,H) might be a viable option *in vivo*. The main inhibitors of NF-κB-p65 expression in each of the tested extracts might be quercetin derivatives and apigenin in EEAL, luteolin in EEAC and caffeic acid in EEAG, as pointed out in previous studies (Gupta et al., 2010). It has been demonstrated that the number of available hydroxyl groups of aglycone and glycosylated polyphenols is related to modulatory effect of these compounds on oxidative stress (Rice-Evans et al., 1996; Csepregi et al., 2016). The presence of hydroxyl group-rich-flavonoids in EEAL, the extract with the highest TFC and TPC (Table 1), and the phenolic nature of predominant bioactive compounds in EEAC and EEAG, respectively might also have contributed to the significant increase in non-enzymatic antioxidant capacity of cancer cells after high extract dose treatment (Figure 7F, 8F) (*P* < 0.001). Considering that both colon carcinoma and melanoma cells are susceptible to oxidative stress, which further damages their already shattered defense systems (Venza et al., 2015; Luput et al., 2017), the modulation of oxidative stress might be one of the focal anti-cancer mechanisms of *Ajuga* sp. extracts in this study. Moreover, the higher values of TPC and TFC in EEAL compared to EEAC and EEAG (Figure 10) can explain why *A. laxmannii* exerted the best biological activity, inhibiting the proliferation of C26 and B16.F10 cells most efficiently at lower doses, probably in a synergistic manner (de Kok et al., 2008).

In conclusion, the results of our study indicated that the overall cytostatic effect of the investigated plant extracts was exerted through strong inhibitory actions on NF-κB-p65, the key molecule involved in the inflammatory response, and via oxidative stress modulatory effects in both murine colon carcinoma and melanoma cell lines. Among the three selected species, *Ajuga laxmannii* elicited the strongest inhibitory action at lower doses on B16.F10 and C26 cancer cell lines, compared to *Ajuga chamaepitys* and *A. genevensis*, due to the richer composition in bioactive polyphenolic compounds. Nevertheless, extended studies on experimental tumor models could shed more light on the anticancer activity of the selected indigenous *Ajuga* sp. extracts. Our results indicated that *A. laxmannii* extract holds the potential to become an additional form of treatment alongside conventional anticancer drugs.

## Author Contributions

V-FR, LV, AT, and MB contributed to conceptualization and design of the study. V-FR, AS, A-MG, AT, and AM performed the experiments. TC, V-FR, and AM performed the statistical analysis. V-FR, AT, and MB wrote sections of the manuscript. All authors contributed to manuscript revision, read, and approved the submitted version.

## Conflict of Interest Statement

The authors declare that the research was conducted in the absence of any commercial or financial relationships that could be construed as a potential conflict of interest.

## Abbreviations

AE: aucubin equivalent
AKT (PKB): protein kinase B
dw: dried weight
EEAC: ethanolic extract from aerial parts of *A. chamaepitys*
EEAG: ethanolic extract from aerial parts of *A. genevensis*
EEAL: ethanolic extract from aerial parts of *A. laxmannii*
ERK: extracellular receptor kinase
GAE: gallic acid equivalent
IC_50_: half maximal inhibitory concentration
IGF-1: insulin-like growth factor 1
MAPK: mitogen-activated protein kinase
MDA: malondialdehyde
MEK: MAPK/ERK kinase
NF-κB: nuclear factor kappa-light-chain-enhancer of activated B cells
OPLS-DA: orthogonal partial least squares discriminant analysis
PI3K: phosphoinositide 3 kinase
PLS: partial least squares regression
PTEN: phosphatase and tensin homolog
Raf: rapidly accelerated fibrosarcoma protein
Ras: rat sarcoma protein
RE: rutin equivalent
ROS: reactive oxygen species
TAC: total antioxidant non-enzymatic capacity
TFC: total flavonoid content
TIC: total iridoid content
TPC: total phenolic content.
